# Implementation of a Substance Use Recovery Support Mobile Phone App in Community Settings: Qualitative Study of Clinician and Staff Perspectives of Facilitators and Barriers

**DOI:** 10.2196/mental.4927

**Published:** 2016-06-28

**Authors:** Sarah Lord, Sarah K Moore, Alex Ramsey, Susan Dinauer, Kimberly Johnson

**Affiliations:** ^1^ Center for Technology and Behavioral Health Department of Psychiatry Geisel School of Medicine at Dartmouth College Lebanon, NH United States; ^2^ Center for Comprehensive Pain Management and Palliative Care Capital Health Medical Center Pennington, NJ United States; ^3^ Washington University School of Medicine Department of Psychiatry Washington University in St. Louis St. Louis, MO United States; ^4^ University of Wisconsin Madison Madison, WI United States

**Keywords:** substance abuse, relapse prevention, mobile apps

## Abstract

**Background:**

Research supports the effectiveness of technology-based treatment approaches for substance use disorders. These approaches have the potential to broaden the reach of evidence-based care. Yet, there is limited understanding of factors associated with implementation of technology-based care approaches in different service settings.

**Objectives:**

In this study, we explored provider and staff perceptions of facilitators and barriers to implementation of a mobile phone substance use recovery support app with clients in 4 service settings.

**Methods:**

Interviews were conducted with leadership and provider stakeholders (N=12) from 4 agencies in the first year of an implementation trial of the mobile phone app. We used the Consolidated Framework for Implementation Research as the conceptual foundation for identifying facilitators and barriers to implementation.

**Results:**

Implementation process facilitators included careful planning of all aspects of implementation before launch, engaging a dedicated team to implement and foster motivation, working collaboratively with the app development team to address technical barriers and adapt the app to meet client and agency needs, and consistently reviewing app usage data to inform progress. Implementation support strategies included training all staff to promote organization awareness about the recovery support app and emphasize its priority as a clinical care tool, encouraging clients to try the technology before committing to use, scaling rollout to clients, setting clear expectations with clients about use of the app, and using peer coaches and consistent client-centered messaging to promote engagement. Perceived compatibility of the mobile phone app with agency and client needs and readiness to implement emerged as salient agency-level implementation facilitators. Facilitating characteristics of the recovery support app itself included evidence of its impact for recovery support, perceived relative advantage of the app over usual care, the ability to adapt the app to improve client use, and its ease of use. The mobile phone itself was a strong motivation for clients to opt in to use the app in settings that provided phones. App access was limited in settings that did not provide phones owing to lack of mobile phone ownership or incompatibility of the app with clients’ mobile phones. Individual differences in technology literacy and provider beliefs about substance use care either facilitated or challenged implementation. Awareness of patient needs and resources facilitated implementation, whereas external policies and regulations regarding technology use introduced barriers to implementation.

**Conclusions:**

The conceptually grounded facilitators and barriers identified in this study can guide systematic targeting of strategies to improve implementation of mobile phone interventions in community treatment settings. Results also inform the design of technology-based therapeutic tools. This study highlights directions for research with regard to implementation of technology-based behavioral health care approaches.

## Introduction

Addiction and mental health treatment programs have been particularly slow to adopt evidence-based practices [1]. Incompatibility of time- and labor-intensive interventions with the realities of care systems presents operational barriers to transfer of evidence-based treatments into practice. Furthermore, a majority (90%) of individuals with substance use disorders do not receive treatment, suggesting that the current care system is either inaccessible or unacceptable to the 21 million Americans who present with substance use disorders annually [2]. For those who receive some form of treatment, the likelihood of relapse is high, particularly if recovery supports are not in place [3].

There is strong and growing evidence to support the effectiveness of technology-based treatment approaches for substance use disorders across the care continuum, including screening and assessment [4,5], treatment [6-15], and recovery support [16]. Such technology-based approaches can be delivered through computers, laptops, or tablets (eg, Web-based treatment for substance use disorders) or by way of mobile phones (eg, addiction recovery support app), either as stand-alone interventions or as augments to care. Studies have consistently demonstrated that technology-based approaches can work as well as, or better than, traditional therapeutic approaches delivered by trained clinicians [11,17,18].

Mobile phone technologies offer a promising platform for delivery of substance use treatment approaches. Use of mobile phone technologies continues to rapidly grow across age, race ethnicity and geography, and consumers increasingly rely on the Internet and mobile phone–based tools for health information [19]. Although disparities in access to mobile phones exist, access is increasing among even the most disadvantaged populations. Approximately 66% of adults in the United States now own a mobile phone, up from 58% in 2014 [20]. Ownership is highest among adults aged 50 years and under (particularly young adults) and lowest among those aged 65 years and older. Ownership is associated with relatively higher education and income levels for those older than 30 years. Mobile phone ownership is most financially tenuous for the subset of users who depend on their mobile devices the most (ie, low-income individuals who use mobile devices as their sole source of Internet access) [20]. Despite these disparities, mobile phone technologies offer the potential for many individuals to access support when they need it the most.

Technology-based substance use treatment approaches offer the potential for on-demand access to care across time and geographic location. These tools can also extend the reach of services to traditionally underserved and disfranchised populations who perceive stigma regarding service use, such as those with substance use conditions or mental illness. There is also growing support for the cost-effectiveness of technology-based substance use treatment approaches [21,22]. The Addiction Comprehensive Health Enhancement Support System (A-CHESS [16]) is a recovery support app for mobile phones. The app was developed to align with therapeutic constructs associated with substance use relapse prevention, including monitoring of use, relevant information about triggers and recovery, skill building and restructuring activities (ie, relaxation exercises, avoidance strategies), and support (eg, on-demand outreach to recovery support people, meeting locator, online peer discussion forum). In a randomized controlled study with clients who had completed residential treatment for alcohol dependence, those who used the mobile phone app demonstrated fewer risky drinking days and higher self-reported abstinence at 6 months relative to those who received standard care [16].

Despite strong empirical evidence to support the effectiveness of technology-based therapeutic approaches to substance use, the field is relatively nascent with regard to guidance on the process of implementing these approaches in community care settings. In this qualitative study, we explored provider and staff perceptions of implementation of the A-CHESS mobile recovery support app with clients in 4 addiction service settings. By identifying facilitators and barriers to implementation, we can begin to develop clearer guidelines to support adoption and implementation of technology-based tools in diverse settings.

We used the Consolidated Framework for Implementation Research (CFIR) [23,24] as an organizing framework for the study. The CFIR represents a unifying typology of implementation models and constructs associated with successful implementation of innovations in health service delivery systems [25-27]. The framework outlines key constructs in 5 domains, including characteristics of the intervention, characteristics of individuals using the intervention, qualities of the organization in which the intervention is implemented and of the broader community–social environment within which organizations operate, and the implementation process itself. The CFIR framework has been used in a number of health service areas, including weight management [28], health information [29], mental health care [30-33], and technology-based approaches to behavioral health care [34]. The framework provided a foundation from which to identify facilitators and barriers to implementation of the A-CHESS mobile phone app with clients in the community service settings.

## Methods

The first author’s institutional review board (IRB) approved the study. Participating service sites were members of the Comprehensive Health Enhancement Support System Health Education Consortium (CHEC), organized by the A-CHESS app development researchers at the University of Wisconsin, Madison (UW). The purpose of the CHEC was to study how A-CHESS would be used by service organizations in naturalistic implementations. As part of consortium membership, agencies made a donation ($10,000) to support consortium activities and agreed to participate in studies generated from consortium member interests, as possible. The UW team provided member agencies with access to A-CHESS and ongoing technical assistance. Technical assistance included training materials for program setup and monthly telephone support.

Eight agencies participated in the first year of the consortium, representing substance use treatment, community behavioral health, and drug court settings located in the Northeast (2), Midwest (2), South (2), and West (2) of the United States. Each agency committed to make A-CHESS available to up to 100 clients over a 1-year period. Agencies determined how and with whom A-CHESS would be used at their organization as well as how to engage clinical and administrative staff in the implementation process.

### Participating Agencies

Dartmouth researchers presented the plan for this implementation process study to the 8 consortium agencies during a regular CHEC monthly teleconference and sent a separate follow-up email invitation to each consortium member. Of the 8 sites, 4 (50%) agreed to participate in this study. Four consortium agencies elected not to participate, primarily owing to lack of time and delays in implementation of A-CHESS. Agencies that participated in the study did not differ from those that did not participate on key demographic indicators, including type of setting, services provided, and client demographics.

All 4 participating organizations were within the first year of implementation of A-CHESS. Agency details include:

1. A northeastern addiction recovery center that specializes in services to veterans used A-CHESS in combination with medication-assisted treatment for veterans with a high rate of alcohol detox admissions. The agency provided mobile phones and data plans to clients, through funding from a federal grant, to foster standardization of client experience and internal technical support. The medical director, nurse case manager, and director of information technology were interviewed for this study.

2. A northeastern drug court program integrated A-CHESS into their substance abuse treatment program for offenders. Mobile phones and data plans were provided to clients through a federal grant. Interviews were conducted with the change leader, the caseworker, and the peer recovery coach hired to support implementation.

3. An addiction treatment center based outside a major northeastern city offered A-CHESS to “alumni” of its inpatient treatment program. Only clients with compatible mobile phones were offered the A-CHESS app. Interviews were conducted with the alumni services coordinator, the inpatient administrator, and the training director involved in implementation.

4. An outpatient behavioral health agency in the Midwest offered A-CHESS as a resource for posttreatment support for clients with mobile phones. Interviews were conducted with the agency program director, training director, and clinical supervisor overseeing implementation of A-CHESS at the agency.

### Stakeholder Recruitment

For each participating agency, 3 stakeholders were invited to participate in the study. Stakeholders represented leadership and clinical perspectives on the implementation of A-CHESS. Because the study focused on agency efforts to promote implementation of the mobile phone app, clients were not included as stakeholders. All stakeholders who were invited agreed to participate.

### Interviews

An interview guide was created to elicit stakeholder perspectives on the implementation process of the A-CHESS mobile phone app. The guide included probes associated with the decision to become a consortium member, preimplementation planning and preparation strategies, implementation experiences, monitoring of progress and success, experiences of technical assistance and support, and plans for sustainability.

### Sample

A total of 12 stakeholders were interviewed for the study (3 from each agency). Participants were 50% female, predominantly white (91%), and ranged in age from 25 to 53 (mean: 36.7) years. A postdoctoral researcher trained in qualitative methods conducted the 20- to 30-minute interviews in early 2013. All interviews were audio-recorded and transcribed, with the exception of one wherein the audio recording failed. In this instance, interview summary notes were created immediately after the interview. Because summary notes were not conducive to further coding, we analyzed 11 interviews for this study.

### Analysis

Researchers trained in qualitative methods (coauthors SM and SL) reviewed and coded interview transcripts using a deductive, consensus-based directed content analysis approach to strengthen the trustworthiness of the analysis [35,36]. Guided by the CFIR model, a coding scheme was developed that outlined each of the constructs to represent either a barrier or facilitator to implementation of the mobile recovery support tool. The coders independently coded each transcript using the coding scheme to document presence of given constructs throughout the narrative and whether a barrier or facilitator. The coders met frequently to ensure that coded text segments were consistent with code definitions; inconsistencies were resolved through discussion to achieve consensus. Coding discrepancies were primarily related to perceived conceptual overlap of CFIR constructs.

## Results

Results are described first in terms of the implementation process for each agency, followed by description of contextual facilitators and barriers to implementation that emerged across agencies and stakeholders. Represented CFIR coding themes across agencies and stakeholders are depicted in Multimedia Appendix 1. Multimedia Appendix 2 includes code conceptualizations and representative quotes.

### Implementation Process

#### Veteran Substance Use Treatment Center

This agency offered a set of integrated services to clients including A-CHESS, naltrexone, individual therapy, and a recovery coach. At the time of the interviews, 45 of 50 eligible clients were using A-CHESS. The agency fostered engagement through creation of a dedicated implementation team that included the following: (1) a nurse case manager who identified and trained eligible clients and (2) the information technology director who set up the technology infrastructure, oversaw all internal technical assistance and troubleshooting, and managed the data collected through A-CHESS.

Implementation of A-CHESS was marked by detailed planning before the launch, including: (1) identifying strategies for recruitment of clients, obtaining consents, and training of all agency providers and clients, (2) identifying the types of mobile phones and data plans that would be most compatible with the agency service area (working with a local phone vendor), and (3) determining processes for monitoring and maintaining online app features (eg, discussion board) and for how to effectively use data to inform implementation. Clearly defined roles, regular meetings, and open communication between team members and with clients allowed for adaptability during implementation to improve compatibility with agency and client needs.

We saw actually a tipping point...one veteran had reached out on the discussion board and there was radio silence…the member of our team managing A-CHESS had a talk with the veterans and the next week, the same veteran sent out a distress signal and 20 vets descended upon the cellphone, and that was the tipping point. Now it’s instant support.

Medical Director

Ongoing collaboration with the UW app development researchers to address technical issues and adaptations as needed to improve fit with the agency and clients also facilitated implementation, as did routine reviews of data to evaluate implementation and client engagement.

… our CEO, he is definitely the one who is…taking the numbers and talking to other places where he thinks this could be beneficial…It could be a grant, it could become a VA benefit…trying to show the VA that it’s gonna cost them a lot less to keep these people in the A-CHESS program than paying for five detoxes a year…inpatient stays are always more expensive.

Director IT

#### Drug Court

A-CHESS and mobile phones were offered to all drug court participants. A dedicated team was created that included a certified change leader, a caseworker, and a peer recovery coach. At the time of the interviews, 40 clients were using the recovery support app. Familiarity with A-CHESS from a prior pilot facilitated preimplementation activities. Careful planning of client training, execution of implementation, and tailoring of the app to meet client needs facilitated implementation. The agency worked closely with the UW researchers and their IRB to address concerns about features that could compromise client privacy and confidentiality (with potential legal implications) to ensure protection of clients, including prohibiting probation officers from accessing client information.

Regular team meetings, open communication between team members and with clients, close attention to client needs, and ongoing review of data to monitor client usage, risk, and outcomes all helped to create an organization culture that valued use of A-CHESS and made it a routine aspect of treatment. Client-centered implementation strategies to promote engagement included staged introduction to new clients and use of peer recovery coaches, establishing contracts with clients to set clear expectations about app usage, encouraging clients to try the app before committing to use it, using consistent client-centered messaging about the app, and supporting peer-driven management of app features (eg, discussion board).

It is client-to-client…our staff step back…and we let the peers run it. They police it…Because we don’t dominate it, they own it. They feel very empowered…

Change Leader

The more people you have using the app, the more benefits others get out of it…You have to encourage clients to use it. Once they start using it…the clients will start encouraging the other clients to use it…Make sure clients know it’s for their benefit, it’s for them. The minute they think it’s for your benefit you will find they are resistant…

Peer Recovery Coach

#### Addiction Treatment Center

The team at this agency offered the A-CHESS app to alumni clients of the inpatient treatment program as one of several postdischarge resources. Access was available only to those with a compatible mobile phone. Inpatient alumni were targeted owing to disinterest in the A-CHESS project among outpatient treatment clinicians. The focus on this client subgroup also allowed for a more manageable implementation process. At the time of the interviews, approximately 40 clients were using the app. 

The agency engaged a dedicated team that included the alumni services coordinator, the inpatient administrator for clinical outreach, and the training director. The team paid careful attention to planning implementation rollout, including designing a course of action for training clients with the app, tailoring implementation for client subgroups (eg, older clients, those with learning disabilities), setting expectations for use of the discussion board, and identifying response strategies to “Panic” outreach. Clients were oriented to the app as part of inpatient discharge planning to build awareness and interest in use of the app as a postdischarge resource.

We always talk about the alumni stuff, and we always talk about the app and just kind of put it in their head that there is this app out there and it’s really cool…After they’ve been there a few weeks, that’s when I start meeting with them and talking about aftercare…and just letting them play around with the app on my phone…Before their discharge we meet again to discuss if they want to get [the app] and then I will actually download

Inpatient Administrator

Other process facilitators included working collaboratively with the UW app development researchers on adaptations to improve client engagement (eg, notification feature for discussion board) and ongoing use of data to monitor client engagement and implementation progress. For example, early review of client usage data and time spent in relapse suggested that relapse times were shorter for those clients more actively using the app, i.e. agency staff was able to intervene quickly because of notification about relapse. Agency stakeholders used this information to promote engagement of clients and to highlight the value of the mobile phone app to administrators to promote ongoing adoption.

#### Community Behavioral Health Agency

This agency offered A-CHESS as an ancillary posttreatment support for clients with a substance use disorder who had compatible mobile phones. At the time of the interviews, 5 clients had been set up to use the recovery support tool. A clinical supervisor was in charge of implementation efforts. The agency was familiar with the app from successful participation in prior projects wherein clients were given mobile phones. In this study, there were challenges recruiting clients with mobile phones. 

Recruitment strategies shifted to target younger college-aged clientele, with limited success. The clinical supervisor also described general plans to make A-CHESS a more central component of treatment planning for clients. Stakeholders did not elucidate specific planning strategies for implementation of A-CHESS, ways in which data could inform implementation, or whether they engaged the UW researchers to promote implementation.

### Facilitators and Barriers to Implementation

Salient barriers and facilitators to implementation of the app across settings and stakeholders are described in the following section (see Multimedia Appendices 1 and 2 for detail).

### Inner Setting (Agency Characteristics)

#### Compatibility: Facilitators

Across settings and stakeholders, perceived compatibility of A-CHESS with client needs emerged as a salient facilitator to implementation. Themes associated with the perceived compatibility included the ability to communicate when clients needed it the most (eg, “clients reach to their phones as a way to interrupt a bad moment” [Veterans Substance Use Treatment]), and to promote client empowerment (eg, “…it puts the power of recovery in the hands of the individual. It is the quintessential strength-based, person-centered model.” [Behavioral Health]

#### Compatibility: Barriers

Noted barriers included app features that were incompatible with client populations. For example, stakeholders from the veterans’ treatment setting noted that the name of the outreach feature (Panic Button) was not compatible with military training (ie, “Soldiers don’t panic”), and was thus a feature this client subgroup was ambivalent about using. Drug Court stakeholders noted that the location-tracking feature could be used to violate client privacy and result in additional legal issues.

Agency-level compatibility barriers included concerns about lack of reimbursement for use of the mobile phone app in the care process, provider resistance to use of technology with clients (eg, concerns about therapeutic boundaries and 24/7 liability), and organization policies restricting use of mobile phones.

#### Other Agency-Level Facilitators

Implementation readiness marked by clear leadership support and availability of resources to support implementation (eg, training, dedicated team) facilitated implementation of A-CHESS with clients. Clear and consistent messaging to staff about the relative priority of the mobile phone app, open communication among staff, and a positive learning climate that supported workflow adaptation to improve implementation all facilitated implementation.

### Intervention Characteristics

Characteristics of the A-CHESS app itself were salient for implementation. Design quality and packaging and evidence of strength and quality were the two most referenced characteristics.

#### Design Quality and Packaging: Facilitators

Design quality and packaging is defined as how the intervention is bundled, presented, and assembled [23,24]. In this analysis, we interpreted “bundling” as the app being inseparable from the mobile phone. The phone itself was a significant perceived benefit (“There is a huge incentive for our clients to have a free phone…” [Drug Court]). Other facilitators included the ability to preprogram important client support contacts to foster easy outreach when needed (“To just be given a phone to say dude, my number is in there, press a number and call me, the ease of access…it just sort of freed them…” [Drug Court]), the online discussion board (“It’s giving them a place to reach out to, a place to vent, a place to feel supported when maybe they can’t get to meetings” [Addiction Treatment]) and features to aid those with literacy challenges, including speech-to-text functionality.

#### Design Quality and Packaging: Barriers

Accessibility was a significant barrier to implementation in agencies that did not issue mobile phones both by virtue of clients not owning mobile phones or having phones that were incompatible with the A-CHESS android app (ie, iPhone or Blackberry). A number of clinical barriers were noted. For example, drug court staff were concerned about adverse effects of relapsing clients’ posts on other clients in the online community. 

A stakeholder from the addiction treatment setting expressed concern about potential iatrogenic triggering of relapse by the location-based alert feature that makes a user “aware of every liquor store in your area.” Liability issues were also noted, including concern about the need for ongoing monitoring and response to client postings on the discussion board in the event of actionable incidents (eg, suicide threats). Other noted barriers included limited flexibility of the online administrative feature for providers, navigation challenges (eg, duplicate login areas) and sporadic technical problems with features that required wireless access (particularly in rural areas), and the influence of rapidly changing technologies.

#### Evidence of Strength and Quality: Facilitators

Positive perceptions about the quality of the mobile phone app as a recovery support tool facilitated implementation across care settings (ie, “We’ve found this to be a great tool to stay connected, and it’s all recovery-based, which is great” [Addiction Treatment]). As a drug court stakeholder noted,

The relapse prevention model of A-CHESS itself is really well demonstrated with clients…the fact that clients can reach out 24/7 to peers…that interrupts the moment…the relapse prevention moment, they had something to do instead of sitting in their own head…it really does play out that clients reach to their phone as a way to interrupt a bad moment.

True to the mobile phone app’s purported mandate—to improve continuing care for individuals with substance use disorders by offering ongoing emotional and instrumental support—these quotes all reference contributions to adaptive functioning.

#### Evidence of Strength and Quality: Barriers

Salient barriers with regard to strength and quality of the app included limited longer term client engagement with the app (“Like a lot of other apps, you use it for a month…and then you start losing touch with it” [Addiction Treatment]) and questions about impact with dissemination to a broader client base (“I think if you have too many people on this…you kind of get lost in the whole cyberspace thing of who is this person—anybody could be on there…you want to keep it small so people feel comfortable actually interacting and putting messages on there…you get more interaction when people know each other” [Addiction Treatment]).

#### Relative Advantage

Perceptions of relative advantage of the recovery support app over usual care also facilitated implementation across service sites, particularly with regard to ongoing client support:

A lot of people who deal with drug issues are pretty solitary people…so it’s hard to get them to open up. When you’re doing it through the application, it’s somewhat anonymous…you’re not standing face-to-face…and it makes it a little bit easier to develop friendship.

Drug Court

Stakeholders generally did not indicate perceived relative advantages of the app with regard to their own workflow (eg, “Does it save me…on paperwork or phone calls? I would not say either. It’s probably just the same” [Addiction Treatment]).

#### Ease of Use

Perceived ease of use of the app was a facilitator to implementation across settings (eg, “It is a very safe app; anything you press it easily directs you to go right back to it” [Addiction Treatment]).

#### Trialability and Adaptability

The ability to try the app and adapt it to improve workflow and client use emerged as important implementation facilitators. For example, to overcome usability barriers with the discussion board due to duplicate login navigation, the implementation teams worked with UW development researchers to improve access to the discussion board and create notifications to alert clients to new material.

#### Cost

Cost was a perceived barrier to sustained implementation in the Veteran Substance Use Treatment Center, Drug Court, and Community Behavioral Health agency. Long-term sustainability of providing mobile phones to clients and paying for technical assistance from the UW development team were the most salient cost concerns.

### Individual Characteristics

Providers’ treatment philosophy and beliefs about the use of technology influenced implementation (eg, “this is not how I work with people”). Individual differences in clients’ technology and reading literacies, and degree of learning disability impacted the level of training required for implementation. The intuitive ease of use of the app aided in self-learning.

Once they got the hang of it they really don’t need more instruction. They loved it once they got playing with it. It was a surprise; people we thought would not use it much did use it

Veterans Substance Use Treatment

### Outer Setting

Overall agency awareness of patient needs and resources was positively associated with implementation. Agencies that modified implementation strategies to improve compatibility with clients’ needs experienced implementation success. The behavioral health agency, which had positive experience with A-CHESS in a prior pilot, referenced unawareness of patient needs and resources, specifically with regard to clients’ relative lack of access to mobile phones. This factor contributed significantly to the agency’s challenges with client recruitment to use the app.

External policies and regulations regarding technology use also introduced barriers to implementation. For example, in the drug court, concerns about client privacy and regulations prohibiting mobile phone use were only circumvented through IRB study provisions and a plan to exclude probation officers from access to client information from the mobile phone app. Such exclusion could be more difficult outside a research context.

**Summary of Implementation Process Strategies**

Planning

- Identify what mobile phones to use (if providing phones) to maximize compatibility with clientele and agency needs
- Develop client recruitment strategies, when and how to introduce the app to clients, and training protocol
- Create clear plans for monitoring program features to ensure client safety and privacy
- Identify indicators of implementation success and develop plan for consistent monitoring and use of data to inform implementation and agency practices

Engagement

- Create a dedicated internal team with clear role and responsibilities to lead implementation
- Identify staff with positive attitudes toward the mobile phone recovery support approach to serve as champions to promote buy-in among clinicians and clients
- As possible, collaborate with the mobile phone app development team to address technical issues and create adaptations to improve client engagement and fit with agency and client needs
- Work with technology vendors to ensure that mobile phones to be issued are compatible with the mobile phone app software, the local service area, and the technology infrastructure of agency
- Orient clients to the app early to build awareness and interest (eg, as a postdischarge resource during inpatient care)
- Use contracts with clients to set mutual expectations about mobile phone app use
- Seed discussion forums with conversation content to build client engagement

Execution

- Scale rollout to work out implementation challenges
- Meet regularly to review implementation and adjust workflow as needed
- Obtain client feedback regarding app experiences to guide implementation adaptations

Reflection/evaluation

- Conduct ongoing review of data to monitor client usage, risk, and client outcomes
- Use data to adapt workflow processes to promote implementation

**Summary of Implementation Strategies by CFIR Context Domains**

Inner Setting Characteristics

- Adapt technology and implementation plans to maximize compatibility with agency workflow and needs
- Train clinicians and staff to promote awareness about the mobile phone app and emphasize priority of the app as routine part of care
- Develop and clearly communicate standards and practices to ensure protection of clients and clinicians with regard to use of the mobile phone recovery support app
- Communicate clear indicators of implementation success to all staff to build perceptions of relative advantages of the technology-based approach and elicit staff engagement to help overcome implementation barriers

Intervention characteristics

- Provide mobile devices to foster standardization of client experience and technical support
- Preprogram important client support contacts to foster easy outreach when needed
- Seek funding (eg, donations, minigrants) and leverage state Medicaid billing codes to subsidize hardware and software purchases
- Trial the mobile phone app with end users to promote buy-in

Characteristics of individuals

- Provide ongoing technical assistance and education to staff and clinicians to increase perceptions of the app as compatible with client needs and effective care
- Train clients individually to accommodate differences in technology literacy and learning disabilities
- Tailor the app to meet client needs
- Tailor implementation for client subgroups
- Highlight features to aid those with literacy challenges (eg, speech-to-text functionality)

Outer setting characteristics

- Maintain client-centered approach to care that prioritizes client needs and resources
- Assess technology ownership and use among an agency’s client base to provide foundation for planning of technology-based service delivery initiatives
- Use data from successful implementation to inform administrative policy decisions
- Change agency policies if necessary to accommodate use of mobile care approaches

Textbox 1 summarizes successful implementation process strategies. Textbox 2 summarizes strategies within each of the CFIR context domains that facilitated implementation.

## Discussion

The CFIR conceptual framework provided a valuable lens through which to identify key barriers and facilitators to implementation of A-CHESS with clients from 4 service settings. Results of the study contribute to the field in 3 substantive ways: (1) informing practice for implementation of mobile phone technology approaches for addiction treatment and behavioral health care more broadly, (2) informing development of mobile phone apps to optimize implementation success, and (3) informing directions for implementation science research with regard to use of mobile phone technologies for behavioral health care.

### Informing Practice

Successful implementation of the recovery support app was marked by careful attention to planning for implementation before launch, including what mobile phones to use, what client populations could benefit the most from program use, when to introduce the app to clients, processes for training staff and clientele, how to monitor program features most effectively in terms of staff time and expertise, and how to effectively use data to inform implementation. A key component to planning was early engagement of a dedicated, appropriately trained internal team to foster implementation buy-in among agency providers and clients and facilitate implementation. Iterative and ongoing technical assistance from the app development team to improve the fit of A-CHESS with the agencies and their respective clientele also facilitated implementation. One agency also worked with a local technology vendor to ensure that the mobile phones to be issued to clients were compatible with the mobile app software, the local service area, and the technology infrastructure of the agency. This pre-implementation preparation paved the way for standardization of client experience and internal technical assistance. The use of internal change agents and external supports to promote implementation in systems of care is central to a number of implementation models [25-27]. This study provides a lens on how agencies used internal staff and outside supports to improve client and agency experiences with the mobile phone recovery support app.

Consideration of client needs was central to implementation and informed adaptations to address subgroup needs (eg, learning disability, literacy, drug court involvement), as well as strategies to support client engagement, such as training all staff about the app so that they would be better prepared to respond to clients’ questions regarding their own experiences with the app. The most successful implementation occurred in agencies that actively created a culture that positioned the mobile phone app as a routine part of relapse prevention care. The community behavioral health agency had the most challenges with implementation in large part due to lack of awareness about the technology resources of its clientele. As the only participating agency that did not focus exclusively on addiction treatment, competing service demands may have made it more difficult to prioritize the mobile phone recovery support app as a central part of care. Assessing technology ownership and use among an agency’s client base would create a foundation from which to plan technology-based service delivery initiatives.

### Facilitators and Barriers to Implementation

#### Agency Level

Perceptions of A-CHESS as compatible with client needs were central to stakeholders’ assessment of implementation. Such compatibility was noted across substance use diagnostic categories, that is, alcohol disorders as well as other substance use disorders (eg, drug court). Compatibility, or fit, of interventions with client and organizational needs is a key aspect of many implementation models [25-27] and is associated with implementation of substance use care in community service settings [37]. Replication studies with clients with primary substance use disorders other than alcohol would strengthen generalizability of the results found here.

Consistent with other research with provider stakeholders in behavioral health care settings [34], perceived compatibility barriers such as concerns about billable time, therapeutic boundaries, privacy, and liability (ie, response to suicide threats after hours) are all important issues to address in planning implementation of technology-based care approaches. As would be the case with introduction of any service innovation, these issues should be addressed according to best practices for clinical care at the agency. Strategies to address concerns include ongoing training and support of clinical staff, clear communication about standards and practices with regard to protection of clients and providers, and ongoing collection and sharing of data regarding implementation success [34]. Ongoing provision of clear indicators of implementation success (and barriers) to staff can foster positive perceptions about the relative advantages of technology-based care approaches and elicit staff engagement to help overcome implementation barriers [23,24]. Establishing an organizational climate that emphasizes the relative priority of the mobile phone approach to care promotes collaboration, communication, and flexibility to adjust course and adapt as challenges emerge.

#### Intervention Level

The potential of mobile phones for enhancing continuing care was salient across agencies and stakeholders. Access to mobile phones was a powerful motivator to client buy-in to use the app in agencies that offered phones for A-CHESS implementation. Cost was a barrier to sustained ability to provide mobile phones to clients. In agencies that did not provide phones, accessibility to the A-CHESS app was limited to those clients that owned app-compatible mobile phones. Fortunately, mobile phone ownership continues to increase across demographics [20]. Still, this study highlights the real continued disparity in mobile phone ownership, particularly among disadvantaged, rural populations.

Strategies to promote sustainable client access to evidence-based mobile apps such as A-CHESS are essential. When possible, agencies can seek funding (eg, donations, mini-grants) and leverage state Medicaid billing codes to subsidize hardware and software purchases [38]. Alternatively, agencies have successfully integrated A-CHESS into their service line and overall business model, establishing a reputation in the field and demand among consumers [38]. Another strategy to reduce costs is to lend mobile phones with the app preinstalled to clients entering treatment and recycle those phones to new clients as previous clients begin to experience diminishing returns from the app. Setting clear expectations with clients at the outset and thoroughly debriefing on retrieval of the phone to outline a clear plan for continued recovery support would be important with this approach. Changing policies in health care could eventually support “prescribing” mobile phones with the installed app as a covered cost.

The online peer discussion forum was the most popular feature of A-CHESS for clients, as reported by stakeholders across the 3 agencies that successfully implemented the mobile phone app. This feature empowered clients and fostered active engagement in their recovery process through opportunities to both receive and offer support to others. The online forum created a way for individuals to connect with others and overcame traditional barriers to in-person recovery support groups, including difficulty finding meetings or inability to attend at scheduled times, and perceived stigma or discomfort with in-person meetings.

There were differences between agencies in the level of monitoring and seeding of the forum by the care teams. Stakeholders from the drug court set expectations for forum use and client privacy protection and encouraged client ownership of the forum; stakeholders from the Veterans and addiction treatment settings were more involved in seeding the forum to encourage client engagement. One way to address concerns that A-CHESS communities might get too large is to create multiple groups as volume increases based on obvious delineations within the community-at-large (ie, separate forums for probationers and post-probationers or for younger and older adults). Research is needed with clients in addiction recovery to broaden our understanding of the role and benefits of online support communities, such as those offered in A-CHESS, for recovery outcomes. Key research questions include when and for whom these online support networks are most helpful and how online communities compare with inperson self-help groups in terms of mechanisms of influence on client outcomes (eg, bonding and support, goal direction and structure, promotion of non-drug–using norms, fostering self-efficacy and coping skills) [39].

#### Individual Characteristics

Variability in implementation was primarily due to provider attitudes regarding use of technology with clientele and to individual differences in technology literacy of clients who required different levels of training. Although achieving buy-in for service innovations from all clinical staff is unlikely at the outset, agencies can optimize buy-in by carefully selecting internal champions as part of implementation team that will promote use of the mobile phone app by clients. Ongoing sharing of data that supports implementation success among clients can help persuade others about the relative advantages of the technology. The intuitive interface design and ease of use of the A-CHESS app allowed clients to easily learn to use the app with practice.

#### Outer Context

Agency policies prohibiting client use of mobile phones and more stringent privacy regulations for particular subpopulations (ie, drug court–involved) can introduce barriers to implementation of mobile substance use treatment technologies. In some cases, liabilities related to specific technology features, such as location tracking, may need to be turned off to proceed with use of the tool for other capacities. In many cases, agency policies can also be changed. Thinking systemically, an innovation can be adapted to improve fit during implementation; organizations can also adapt to accommodate the innovation [40,41]. Key to any policy change is use of data to demonstrate value, such as improving clients’ health and wellbeing and promoting health service quality and efficiency. Furthermore, organizations are encouraged to adapt the innovation to the needs of the agency and client subgroups. Although efforts should be made by people at adoption sites to maintain the core elements of the intervention [42], scholars are increasingly rejecting the assumption that an intervention will yield diminished benefit for clients after being modified to fit real-world delivery settings [43].

#### Postlude: Sustainability

The focus of this implementation process study was a single point in time relatively early in implementation. In a separate study of A-CHESS sustainability among consortium members, 3 agencies had sustained use of the recovery support tool at 24 months, 2 of which were agencies studied here (Veterans Substance Use Treatment and Addiction Treatment) [38]. It may be that the salient barriers noted in implementation for the 2 nonsustaining agencies (Drug Court: cost, client privacy, mobile phone use policies; Behavioral Health: mobile phone accessibility, cost) reduced perceptions of value added by the mobile recovery support tool in an ongoing service package. Issues of sustainability were foremost on the minds of stakeholders in the Veterans’ agency from the outset of implementation. This agency integrated the cost of mobile phones for clients into their overall business model, which contributed to sustainability. The addiction treatment agency experienced early implementation success with a carefully targeted client subgroup.

### Informing Development

Study results can guide developers to create mobile applications to optimize implementation success. A-CHESS was developed based on evidence-based relapse prevention practices, and evidence of these elements promoted implementation. To optimize adoption, features should be developed to align with evidence-based practices, target audience needs and characteristics, and organizational workflow. To the degree possible, apps should be developed to be cross-platform and Web-accessible. User-centered design practices that include iterative feedback from target end users during the development process help ensure that the app is easy to use and can promote client engagement. Ongoing evaluation of data collected from technology-based approaches can help agencies monitor client engagement related to their care and implementation progress. The way in which important implementation data from technology-based care approaches are displayed to stakeholders is often underappreciated. Improving visualization of data to make it accessible and meaningful to stakeholders is critical.

### Informing Implementation Science

This study also highlights directions for future research with regard to implementation frameworks for technology-based care approaches. A challenge to achieving code consensus using the CFIR framework largely reflected overlap of constructs within and between domains as applied to the mobile phone recovery support app (eg, compatibility of the mobile app for clients as a characteristic of the intervention; compatibility with provider practices as characteristic of the organization). The CFIR model also positions implementation as a traditional unidirectional process—an intervention is delivered to clients by providers or clinicians in a given setting. However, in the case of mobile treatment approaches, the mobile phone itself is a context for implementation, as is the sociocultural environment in which mobile apps are used. Implementation frameworks such as the CFIR need to be expanded or adapted to align with the capacities and multidimensional, dynamic nature of technology-based treatment approaches that actively engage clients at various stages of motivation and treatment and expand care beyond clinic walls [44]. Research in the application of the CFIR model as it pertains to technology-based behavioral health treatment tools more broadly is needed.

### Conclusions

Moving science to service is inherently an active process and the implementers, those people who put an innovation to use, are active recipients of these innovations [45]. In the case of mobile phone interventions, such as A-CHESS, there are a number of implementers. Clients literally have the intervention in the palm of their hands to use as they wish *,* and agency leaders and clinicians are in the important role of facilitating implementation by offering the tool to clients, supporting their use of it, and identifying ways that the technology can be sustainably integrated into care delivery more broadly.

There are several limitations to this implementation process study. First, the study was conducted in 4 settings, the sample size was small and only reflected the perspectives of provider and staff stakeholders, and data were collected at a single point in time. Future efforts to explore implementation would benefit from longitudinal data collection from a larger, more diverse range of settings and stakeholders, including those who decided not to use the technology. Second, although providers and staff provide a valuable lens on implementation from the perspective of the larger client base and organization, clients’ experiences with the app would lend valuable perspective on strategies for implementation and ongoing engagement with the app and should be a focus of future research. Finally, it is possible that the relationships between the consortium participants and the UW-Madison team may have inflated the overall impressions of the tool owing to the collective investment in the consortium itself. This possibility is countered by the fact that 2 of the 4 agencies did not sustain use of A-CHESS as demonstrated in later work. Despite these limitations, the study yielded important findings that may generate further research and aid in practice-based efforts to implement mobile substance use treatment approaches.

## Appendix

Multimedia Appendix 1

Multimedia Appendix 2

## Abbreviations

A-CHESS: Addiction-Comprehensive Health Enhancement Support System
CFIR: Consolidated Framework for Implementation Research
CHEC: Comprehensive Health Enhancement Support System Health Education Consortium
IRB: institutional review board

## References

[1] Glasner-Edwards S, Rawson R (2010). Evidence-based practices in addiction treatment: review and recommendations for public policy. Health Policy.

[2] (2013). Results from the 2012 National Survey on Drug UseHealth: summary of national findings. NSDUH Series H-46, HHS Publication No. (SMA) 13-4795. Rockville, MD.

[3] Carroll K (1996). Relapse prevention as a psychosocial treatment: A review of controlled clinical trials. Experimental and clinical psychopharmacology.

[4] Lord S, Trudeau K, Black R, Lorin L, Cooney E, Villapiano A, Butler Stephen F (2011). CHAT: development and validation of a computer-delivered, self-report, substance use assessment for adolescents. Subst Use Misuse.

[5] Butler S, Budman S, Goldman R, Newman F, Beckley K, Trottier D, Cacciola J S (2001). Initial validation of a computer-administered Addiction Severity Index: the ASI-MV. Psychol Addict Behav.

[6] Budney A, Fearer S, Walker D, Stanger C, Thostenson J, Grabinski M, Bickel Warren K (2011). An initial trial of a computerized behavioral intervention for cannabis use disorder. Drug Alcohol Depend.

[7] Carroll K, Ball S, Martino S, Nich C, Babuscio T, Nuro K, Gordon Melissa A, Portnoy Galina A, Rounsaville Bruce J (2008). Computer-assisted delivery of cognitive-behavioral therapy for addiction: a randomized trial of CBT4CBT. Am J Psychiatry.

[8] Carroll KM, Ball SA, Martino S, Nich C, Babuscio TA, Rounsaville BJ (2009). Enduring effects of a computer-assisted training program for cognitive behavioral therapy: a 6-month follow-up of CBT4CBT. Drug Alcohol Depend.

[9] Carroll K, Kiluk B, Nich C, Gordon M, Portnoy G, Marino D, Ball SA (2014). Computer-assisted delivery of cognitive-behavioral therapy: efficacy and durability of CBT4CBT among cocaine-dependent individuals maintained on methadone. Am J Psychiatry.

[10] Nunes E, Matthews A, Stitzer M, Miele G, Polsky D, Turrigiano E, Walters S, McClure EA, Kyle TL, Wahle A, Van Veldhuisen P, Goldman B, Babcock D, Stabile PQ, Winhusen T, Ghitza UE, Campbell Aimee N C (2014). Internet-delivered treatment for substance abuse: a multisite randomized controlled trial. Am J Psychiatry.

[11] Marsch L, Guarino H, Acosta M, Aponte-Melendez Y, Cleland C, Grabinski M, Brady Ronald, Edwards Joyce (2014). Web-based behavioral treatment for substance use disorders as a partial replacement of standard methadone maintenance treatment. J Subst Abuse Treat.

[12] Ondersma S, Svikis D, Schuster C (2007). Computer-based brief intervention a randomized trial with postpartum women. Am J Prev Med.

[13] Ondersma S, Svikis D, Thacker L, Beatty J, Lockhart N (2014). Computer-delivered screening and brief intervention (e-SBI) for postpartum drug use: a randomized trial. J Subst Abuse Treat.

[14] Kay-Lambkin F, Baker A, Lewin T, Carr V (2009). Computer-based psychological treatment for comorbid depression and problematic alcohol and/or cannabis use: a randomized controlled trial of clinical efficacy. Addiction.

[15] Kay-Lambkin F, Baker A, Kelly B, Lewin T (2011). Clinician-assisted computerised versus therapist-delivered treatment for depressive and addictive disorders: a randomised controlled trial. Med J Aust.

[16] Gustafson D, McTavish F, Chih M, Atwood A, Johnson R, Boyle M, Levy Michael S, Driscoll Hilary, Chisholm Steven M, Dillenburg Lisa, Isham Andrew, Shah Dhavan (2014). A smartphone application to support recovery from alcoholism: a randomized clinical trial. JAMA Psychiatry.

[17] Marsch L, Dallery J (2012). Advances in the psychosocial treatment of addiction: the role of technology in the delivery of evidence-based psychosocial treatment. Psychiatr Clin North Am.

[18] Noar S, Black H, Pierce L (2009). Efficacy of computer technology-based HIV prevention interventions: a meta-analysis. AIDS.

[19] Pew Research Center.

[20] Smith A Pew Research Center.

[21] Smit E, de VH, Hoving C, Evers Silvia M A A (2013). Cost-effectiveness and cost-utility of Internet-based computer tailoring for smoking cessation. J Med Internet Res.

[22] Warmerdam L, Smit F, van SA, Riper H, Cuijpers P (2010). Cost-utility and cost-effectiveness of internet-based treatment for adults with depressive symptoms: randomized trial. J Med Internet Res.

[23] Damschroder L, Aron D, Keith R, Kirsh S, Alexander J, Lowery J (2009). Fostering implementation of health services research findings into practice: a consolidated framework for advancing implementation science. Implement Sci.

[24] Damschroder L, Hagedorn H (2011). A guiding framework and approach for implementation research in substance use disorders treatment. Psychol Addict Behav.

[25] Rogers EM (2004). A prospective and retrospective look at the diffusion model. J Health Commun.

[26] Greenhalgh T, Robert G, Macfarlane F, Bate P, Kyriakidou O (2004). Diffusion of innovations in service organizations: systematic review and recommendations. Milbank Q.

[27] Greener J, Simpson D, Lehman Wayne E K (2002). Assessing organizational readiness for change. J Subst Abuse Treat.

[28] Damschroder L, Lowery J (2013). Evaluation of a large-scale weight management program using the consolidated framework for implementation research (CFIR). Implement Sci.

[29] Abbott PA, Foster J, Dykes PC, Marin Heimar de Fatima (2014). Complexity and the science of implementation in health IT--knowledge gaps and future visions. Int J Med Inform.

[30] Briand C, Menear M (2014). Implementing a continuum of evidence-based psychosocial interventions for people with severe mental illness: part 2-review of critical implementation issues. Can J Psychiatry.

[31] Cabassa L, Gomes A, Lewis-Fernández Roberto (2015). What would it take? Stakeholders' views and preferences for implementing a health care manager program in community mental health clinics under health care reform. Med Care Res Rev.

[32] Ditty M, Landes S, Doyle A, Beidas R (2015). It Takes a Village: A Mixed Method Analysis of Inner Setting Variables and Dialectical Behavior Therapy Implementation. Adm Policy Ment Health.

[33] Murphy A, Gardner D, Kutcher S, Martin-Misener R (2014). A theory-informed approach to mental health care capacity building for pharmacists. Int J Ment Health Syst.

[34] Ramsey A, Lord S, Torrey J, Marsch L, Lardiere M (2016). Paving the Way to Successful Implementation: Identifying Key Barriers to Use of Technology-Based Therapeutic Tools for Behavioral Health Care. J Behav Health Serv Res.

[35] Hsieh H, Shannon S (2005). Three approaches to qualitative content analysis. Qual Health Res.

[36] Shenton A (2004). Strategies for ensuring trustworthiness in qualitative research projects. Education for Information.

[37] Patterson S, Weaver T, Agath K, Albert E, Rhodes T, Rutter D, Crawford Mike (2009). 'They can't solve the problem without us': a qualitative study of stakeholder perspectives on user involvement in drug treatment services in England. Health Soc Care Community.

[38] Ford JH, Alagoz E, Dinauer S, Johnson Ka, Pe-Romashko K, Gustafson Dh (2015). Successful Organizational Strategies to Sustain Use of A-CHESS: A Mobile Intervention for Individuals With Alcohol Use Disorders. J Med Internet Res.

[39] Moos R (2008). Active ingredients of substance use-focused self-help groups. Addiction.

[40] Dearing J (2009). Applying Diffusion of Innovation Theory to Intervention Development. Res Soc Work Pract.

[41] Torrey W, Bond G, McHugo G, Swain K (2012). Evidence-based practice implementation in community mental health settings: the relative importance of key domains of implementation activity. Adm Policy Ment Health.

[42] Chorpita B, Becker K, Daleiden E (2007). Understanding the common elements of evidence-based practice: misconceptions and clinical examples. J Am Acad Child Adolesc Psychiatry.

[43] Chambers D, Glasgow R, Stange K (2013). The dynamic sustainability framework: addressing the paradox of sustainment amid ongoing change. Implement Sci.

[44] Litvin E, Abrantes A, Brown R (2013). Computer and mobile technology-based interventions for substance use disorders: an organizing framework. Addict Behav.

[45] Fixsen D, Blase K, Naoom S, Wallace F (2009). Core Implementation Components. Research on Social Work Practice.

