# Supplementary material for: Implementation of a Substance Use Recovery Support Mobile Phone App in Community Settings: Qualitative Study of Clinician and Staff Perspectives of Facilitators and Barriers
Source: JMIR Ment Health. 2016 Jun 28;3(2):e24. doi: 10.2196/mental.4927 (PMC4942682; doi:10.2196/mental.4927)
Supplement: Multimedia Appendix 1 [file mental_v3i2e24_app1.pdf]

Multimedia Appendix 1. Representation of CFIR Domains and Constructs Across Care Settings and Stakeholders

	COMMUNITY SETTINGS										
	VETERAN SUBSTANCE USE TREATMENT			DRUG COURT PROGRAM			ADDICTION TREATMENT		COMMUNITY BEHAVIORAL HEALTH		
	DirectorMedical	IT Director	agerNurse Case	Change Leader	Case Worker	nPeer Recovery	ervices Alumni	linical Inpatient	irectorProgram	DirectorTraining	pervisorClinical
**Implementation Process**											
Planning: Design a Course of Action											
Facilitator	x	x	x	x			x	x			
Barrier					x						
Planning: Needs and Perspectives Considered											
Facilitator											
Barrier											
Planning: Strategies Tailored for Subgroups											
Facilitator	x			x	x		x	x			x
Barrier											x
Engagement/Internal Change Agent											
Facilitator	x	x	x	x	x	x		x			
Engagement/External Change Agent							x				
Facilitator	x	x		x	x	x	x	x			
Execution											
Facilitator	x		x								
Reflection/Evaluation											
Facilitator	x	x	x	x	x		x	x			
Barrier	x	x	x								
**Characteristics of Organization Inner Setting**											
Implementation Climate: Compatibility											
Facilitator	x	x	x	x	x	x	x	x			
Barrier	x	x		x	x			x	x		x
Implementation Climate: Relative Priority											
Facilitator	x	x	x	x			x				
Barrier									x		x
Implementation Climate: Goals and Feedback											
Facilitator											

Barrier											
Implementation Climate: Tension for Change											
Facilitator											
Implementation Climate: Learning Climate											
Facilitator		**x**						**x**			
Networks and Communications											
Facilitator		**x**		**x**				**x**			
Barrier											
Culture											
Facilitator						**x**					
Barrier											
Implementation Readiness: Available Resources											
Facilitator	**x**	**x**	**x**	**x**	**x**	**x**	**x**	**x**			
Barrier			**x**								
Implementation Readiness: Leadership support											
Facilitator	**x**			**x**			**x**	**x**	**x**		
Barrier											
**Intervention Characteristics**											
Design Quality and Packaging											
Facilitator	**x**	**x**	**x**	**x**	**x**	**x**	**x**				
Barrier		**x**	**x**	**x**	**x**	**x**	**x**	**x**	**x**		**x**
Evidence of Strength & Quality											
Facilitator	**x**		**x**	**x**	**x**	**x**	**x**		**x**		**x**
Barrier							**x**	**x**	**x**		
Relative Advantage											
Facilitator	**x**			**x**	**x**	**x**					
Barrier							**x**				
Ease of Use											
Facilitator					**x**	**x**	**x**		**x**		**x**
Barrier		**x**	**x**								
Cost											
Facilitator				**x**							
Barrier		**x**		**x**	**x**	**x**			**x**		**x**
Adaptability											
Facilitator	**x**	**x**			**x**	**x**	**x**	**x**			
Barrier											
Trialability											
Facilitator		**x**		**x**	**x**	**x**	**x**				
Barrier											
Intervention Source											
Facilitator											
Barrier							**x**	**x**			

**Characteristics of Individuals**											
Knowledge and Beliefs about the Intervention											
Facilitator	**x**	**x**	**x**	**x**	**x**	**x**	**x**		**x**		
Barrier		**x**	**x**	**x**			**x**	**x**			
Self-Efficacy											
Facilitator	**x**							**x**			
Barrier											
Access to knowledge and information											
Facilitator											
Barrier											
**Characteristics of External Setting**											
Awareness Patient Needs and Resources											
Facilitator	**x**	**x**		**x**		**x**	**x**	**x**			
Barrier									**x**		**x**
External Policies											
Facilitator					**x**						
Barrier				**x**	**x**						
