# Supplementary material for: Implementation of a Substance Use Recovery Support Mobile Phone App in Community Settings: Qualitative Study of Clinician and Staff Perspectives of Facilitators and Barriers
Source: JMIR Ment Health. 2016 Jun 28;3(2):e24. doi: 10.2196/mental.4927 (PMC4942682; doi:10.2196/mental.4927)
Supplement: Multimedia Appendix 2 [file mental_v3i2e24_app2.pdf]

Multimedia Appendix 2. Domain construct themes and quotes

**CFIR DOMAIN/** Component<br><br>**(Valence)**	**CONCEPTUALIZATIONS/ THEMES**	**REPRESENTATIVE QUOTES**
**P***<br><br>Plan: Design a Course of Action **(Facilitator)**	e.g., screen for hardware ownership, consider timing of phone/app issuance, as well as training<br><br>e.g., how to involve and monitor users; who will monitor users<br><br>Plans to reduce costs: e.g., order all same phones to save on training/technical support time; budget for replacement cost of damaged phones	*'We tweaked our engagement process…when we're doing initial screenings and talking about different services we offer, we also screen to get some data on like if the person has a smartphone,'*<br><br>*'Apparently it really hung things up that we had a drug court criminal justice population and there was a lot of concerns about some inappropriate access or that a jeopardy could happen to the clients based on what they shared. So for our implemented plan, it is strictly clients and clinical staff [with access] and there is a clear boundary and rule that what happens in A-CHESS stays in A-CHESS but clinicians will still respond around safety issues…if somebody is indicating that they are at risk form a homicide suicide they know we're going to respond.*<br><br>*'We built in replacement costs in case we had to – we knew we were probably going to have to replace 50% of the phones was our experience so we built that in.'*
**P**<br>Plan: Strategies Tailored for Sub-Groups **(Facilitator)**	Efforts made to tailor strategies to specific sub-groups (e.g., learning disabled, drug court, older clients)	*'Learning disabilities and literacy is an issue. What we did was show them on our phone, thank goodness there is a voice to text program.'*<br><br>*'What we did was discuss with the Wisconsin people, let's offer a web version…they decided okay that would be acceptable. Those that are 35 – 64 would be more apt to use it on their laptop in the web browser than on their phone app.'*
**P**<br>Engagement: Internal Change Agent **(Facilitator)**	References to role as it relates to the mobile recovery application implementation	*'I was the medication nurse on our detox unit, and when they decided to do this outpatient program, they brought me in to identify the people who meet the criteria to be in the program…Once I identified those people…we gave them their phones, and then I taught them how to use the phone…the app as well.'*
**P**<br><br>Engagement: External Change Agent **(Facilitator)**	Perceptions of the lengths to which the researchers/developers influenced the implementation by working closely with the different agencies to improve compatibility of the mobile recovery support application with agencies and clientele	*'Probation doesn't have access to it specifically for that reason because the UW attorneys said that they didn't want probation to have access to it because they didn't want individuals sanctioned or punished in any way, shape or form for expressing whatever they express on A-CHESS.'*
**P**	Perceptions that implementation of the app	*'I know there's a couple of things that we want to add to either the app or to the phone itself... But for the most part, I would*

Executing **(Facilitator)**	was uneventful and largely without much alteration to plans.	*say we haven't made changes to it.'*
**P**<br><br>Reflecting/ Evaluating **(Facilitator)**	Use data to inform implementation process	*'We went back and looked at patients who were active on the app and suffered a relapse. The time they spent in the relapse and the time it took to get back in treatment was really positive results…you can shorten that time period [relapse to care] by engagement'*
**P**<br><br>Reflecting/ Evaluating **(Barrier)**	Expressions indicating a lack of familiarity with process or outcome data related to implementation.	*'If you have any questions about tracking data or any numbers or anything like that, [Leader] would be the person to talk to about that.'*
**IC**<br><br>Design, Quality and Packaging **(Facilitator)**	The packaging of the recovery support app in a mobile phone was a facilitator for clients and agency staff –	*'There's a huge incentive for our clients to have a free phone.'*<br><br>*'Everyone wanted [A-CHESS]. I mean they still use [the app] for sure, but [the phone] was very helpful for people that didn't have rides, or didn't have phones, and for us to communicate.'*
**IC**<br><br>Design, Quality and Packaging **(Barrier)**	Access: Access to the mobile recovery support app due to -agency clientele' not owning smartphones, not owning phones that supported the app, or agency policy prohibiting use of phones while in treatment	*'We overestimated the amount of people…that [had] a smartphone…'*
	Clinical Care: Perceptions that intervention as packaged introduced challenges to good clinical care and increased liability	*'If they're not engaged, yeah, we can take the phone away [but with one guy] he just went off. He was really drunk and everyone was like, 'Why does he have a phone?' We [couldn't] find him. I can't take his phone away.'*<br><br>*'Even though clinicians will respond in off hours if they can, we don't guarantee it…Someone could say, you know I said I was suicidal at 1:00 in the morning and no one responded. Now that person kills themself and someone finds their phone and says oh, they texted [us] and now they're dead, what happened?'*
**IC**<br><br>Evidence of Strength and Quality **(Facilitator)**	When acknowledging the strength and quality of the mobile recovery support app, general assessments were offered with few details other than reference to addictions or relapse prevention.	*'I think this is a very empowering process…that resonates with the recovery community in general…Particularly with people in recovery it is a specifically good tool'*<br><br>*'We've found this to be a great tool to stay connected, and it's all recovery-based, which is great.'*<br><br>*'The relapse prevention model itself of A-CHESS is really well demonstrated with our clients who say, 'if I waited until my appointment Tuesday at 3:00 to deal with my cravings, I'd be in jail. The fact that I can reach out 24/7 to my peers, that at 3 am I am craving but somebody else is up'…that interrupts that moment. I think that was part of the theory but it really does play out that the clients reach to their phones as a way to interrupt a bad moment.'*
**IC**	Concerns about basic feature	*'Use of the panic button can be a little touchy,' '[the Meeting*

Evidence of Strength and Quality **(Barrier)**	functionality, especially regarding the support outreach and online discussion board.	*<u>Finder</u>] hasn't worked since we've had it,' 'I don't know if <u>the GPS function</u> even works,' '[<u>the Discussion Board</u>] is a separate thing to log into… to go someplace else could be a barrier,' and 'you don't ever have guaranteed confidentiality.'*
**IC**<br><br>Ease of Use **(Facilitator)**	Perceived ease of training to use/learning to use, and safe use	*'It is easy to pick up…Once people get the gist of what its about, its basically them fooling around with it themselves.'*
**IC**<br><br>Ease of Use **(Barrier)**	Perceptions of the complexities of both hardware and software features	*'There were barriers in getting them to even get used to the touchscreen, how to use a smartphone, and its capabilities, and how to get back somewhere."*<br>*'There's a learning curve not just for the patients but also for the staff.'*
**IC**<br><br>Relative Advantage **(Facilitator)**	General belief that the mobile recovery app benefits clients in ways superior to current practice (e.g., improved communication, peer support, making treatment 'fun.'	*'Before we started using it, clients had a hard time keeping in touch with each other.'*
**IC**<br><br>Relative Advantage **(Barrier)**	Do not perceive advantages relative to other approaches to client engagement, workflow, and other innovations.	*'In terms of my actual job, does it save me less on paperwork or making more phone calls? I wouldn't say either – its probably just the same.'*
**IC**<br><br>Cost **(Facilitator)**	Perceptions that costs associated with app implementation were deemed acceptable once clients and staff had exposure to benefits.	*'Obviously its less expensive…If I've got peers helping each other at 3 am, they are not calling emergency services. I don't have a clinician on call.'*
**IC**<br><br>Cost **(Barrier)**	Perceptions that costs associated with app implementation were deemed unacceptable	*'Right now we give them a phone and we pay the plan and obviously that is not sustainable. And we also pay to be a part of A-CHESS and the research consortium and that is not sustainable for us'*
**IC**<br><br>Adaptability **(Facilitator)**	Examples of how the mobile recovery support app was adapted to meet local needs.	*'We had to put an alert on it [discussion board] because people were writing on it, and unless you actually went in to check it, we weren't realizing that people were writing on it. So we changed it so an alert will come on the phones [going] to all of the staff email.'*
**IC**<br><br>Trialability **(Facilitator)**	Examples of how the mobile recovery support app was piloted through scaled roll-outs, targeting small patient subpopulations	*'Encourage a clients to use it. Once they start using it…the clients will start encouraging the other clients to use it…'*
**IS**<br><br>Compatibility **(Facilitator)**	<u>Communication:</u> The non-face-to-face nature of the app-based discussion board, as well as the instant community in the form of a phone pre-loaded with community members and/or friends.	*'A lot of people with drug issues are pretty solitary people. They come into these programs and they're not used to having individuals that they can discuss things with. So it's hard to get them to open up. When you're doing it through the application, it's somewhat anonymous. They know that you know who they are, but you're not standing face-to-face looking at the person and it just makes it a little bit easier to develop friendship.'*

	<u>Empowerment:</u> Enthusiasm for how A-CHESS has the potential to put recovery back in the control of the client – encourages self-determination.	*'[Clients] really enjoyed it. It was fun. A real general thing is an app like that can make treatment fun... Addiction treatment - its not always fun for obvious reasons. [A-CHESS] has really changed that. The other part about it is that it puts the power of recovery in the hands of the individual. It's the quintessential, strengths-based person centered model. Like any app that goes on your phone, you're the captain of whatever it is your doing.'*
**IS**<br><br>Compatibility<br>**(Barrier)**	Organizational focus on lack of compatibility from agency, as opposed to client perspective, e.g., discovering that some staff time engaging with clients on the mobile support recovery application was not billable, clinician resistance to use of technology to engage clients, debates about how to monitor the chat room in terms of liability issues, or simply long-standing agency policies restricting phones	*'We don't' allow our patients to have cell phones…should we allow [patients] to sort of play with it and it was like no that's opening up a whole can of worms. So for our agency that was tough.'*
**IS**<br><br>Relative Priority<br>**(Facilitator)**	Expressions of the pan-applicability of the tool and its use.	*'<u>Every</u> new client got a phone.'*<br><br>*'We <u>always</u> talk about the app and just put it in their head that there's this app out there and its really cool.'*<br><br>*'We <u>all</u> have it on our phone.'*
**IS**<br><br>Relative Priority<br>**(Barrier)**	Expressions of a lack of the perceived relative importance or priority of the app compared with the status quo.	*'One of our clinicians said, 'nope, I'm not going to do it [use the app]. I am comfortable in my clinical training. That is not how I work with people.'*
**IS**<br><br>Networks/<br>Communications<br>**(Facilitator)**	Expressions about the development and immediacy of staff community support with introduction of the mobile support recovery application	*'The nice thing is – like I said – we're a pretty close-knit group, so when questions did come up, all of them were more than comfortable either to call me or ask one of their peers. So, even that seemed to go pretty smoothly for us.'*
**IS**<br><br>Goals and Feedback<br>**(Facilitator)**	General perceptions that client' usage of the app can be 'tracked' and that unless usage can be demonstrated, clients will not see benefits to themselves AND funding will not continue.	*'We keep very accurate records on who's using what, who's availing themselves of the offerings of the menu of the program. What we're finding is that even if some vets have fallen off…they are still doing their weekly surveys, which means they are still connected.'*
**IS**<br><br>Available Resources<br>**(Facilitator)**	Training and staffing as facilitator themes	*'Our IT specialist ran a series of trainings to update us and train us on the use of the application…We were all trained on the use of the application.'*
**CI**		*'[Clients] loved it once they got playing with it,' 'I learned what I learned fooling around with it too.'*

Knowledge and Beliefs about the Intervention **(Facilitator)**	Positive perceptions of intervention	
**CI**<br><br>Knowledge and Beliefs about the Intervention **(Barrier)**	Negative perceptions of intervention	*'One of our clinicians, who is very anti-technology, believes it will ruin her quality of life and spirituality [said] nope, I'm not going to do it…that is not how I work with people.'*
**OS**<br><br>Awareness of Patient Needs and Resources **(Facilitator)**	Larger organizational context positively influence implementation	*'We usually wait two weeks to make sure they adjust well to the program and then we issue a phone'*<br><br>*'You want to grow it and have a lot of people, [but] at the same time, you want to keep it small so people feel comfortable actually interacting and putting messages on there'*
**OS**<br><br>External Policy and Incentives **(Facilitator)**	Larger organizational context positively influence implementation	*'Make sure clients know it's for their benefit, it's for them. The minute they think you're using it for your benefit you will find they are resistant…Let them know, it's solely to benefit you, it's for you…They're a lot more receptive'*
**OS**<br><br>External Policy and Incentives Outer Setting **(Barrier)**	Larger organizational context impede implementation	*'The one systemic barrier…is that we're a very large agency and our chief compliance officer came out with a rule disallowing social media, text and email communication with clients…all of our contacts have to be face-to-face and billable. They had to change our agency policy to allow programs to have funded services for this project…If the grant goes away and we don't have the program, our ability to text clients goes away and it's because of HIPAA and confidentiality…and it's not a documented service. For the professional clinical world, they're still not comfortable with it because it's still kind of out there'*<br><br>*'We were really surprised how few people have smartphones'*
**P** = Process /**IC** = Intervention Characteristics/ **IS** = Inner Setting/ **CI** = Individual Characteristics/ **OS**=Outer Setting		
